# The Novel Potentiometric Approach to Antioxidant Capacity Assay Based on the Reaction with Stable Radical 2,2′-diphenyl-1-picrylhydrazyl

**DOI:** 10.3390/antiox11101974

**Published:** 2022-10-01

**Authors:** Elena Gerasimova, Elena Gazizullina, Sofya Kolbaczkaya, Alla Ivanova

**Affiliations:** Analytical Chemistry Department, Institute of Chemical Engineering, Ural Federal University, 19 Mira Str., 620002 Ekaterinburg, Russia

**Keywords:** 2,2′-diphenyl-1-picrylhydrazyl, potentiometry, antioxidant capacity

## Abstract

For the first time, new possibilities of using the DPPH^•^ as a signal-forming oxidant molecule with potentiometric detection are shown. The CV method confirmed the presence of a quasi-reversible potential-determining system DPPH^•^/DPPH-H under experimental conditions. This fact makes it possible to use DPPH^•^ as the model of the oxidizing agent for obtaining an analytical signal by the potentiometry method. The potentiometric approach makes it possible to obtain the value of the Nernst slope and the antioxidant capacity in one experiment. It consists of an antioxidant supplement and two consecutive DPPH^•^ supplements. In this case, the calculation of the Nernst slope is carried out by introducing the second addition of the oxidizing agent and constructing a calibration curve against the reaction background with an antioxidant. Solutions of individual antioxidants α-tocopherol, quercetin, (±)-catechin hydrate, and α-lipoic acid were studied by the developed approach. A high correlation with the results of spectrophotometric measurements is shown. At the same time, the potentiometry method is devoid of the concentration limitations of the spectrophotometric method, which was confirmed. In the study of plant materials extracts, a high correlation of antioxidant capacity, obtained by potentiometric and spectrophotometric methods, was shown only for objects whose color did not contribute to the DPPH^•^ absorption. The versatility of the potentiometric method for studying objects of any color was shown.

## 1. Introduction

The free radical 2,2-diphenyl-1-picrylhydrazyl (DPPH^•^) is one of the few stable N-centered organic radicals due to the delocalization of the radical in aromatic rings [1]. As a result, it has become widely used to study the antioxidant/antiradical properties of substances. It has a deep purple color, which is widely used in the spectrophotometry. The DPPH^•^, interacting with antioxidants, transforms into a reduced diamagnetic form with a pale-yellow color [2,3]. The rate of decrease of the optical density at the maximum absorption of the radical can be used to evaluate the antioxidant/antiradical properties of individual compounds, added to the solution, and their complex mixtures. There are a large number of modifications of this spectrophotometric approach at present [4,5,6]. Since the DPPH^•^ has paramagnetic properties, the use of molecules in the EPR spectroscopy is sufficiently developed, including for the study of the antioxidant properties of various objects [7,8]. At the same time, the use of spectroscopic methods is limited by the range of determined concentrations and the color of the studied solutions, and the EPR methods are very expensive.

The possibilities of using the DPPH^•^ as a signal molecule in instrumental methods are much wider than described in the literature, despite the widespread use of the DPPH^•^ in spectroscopic methods. This is especially true of electrochemical methods, which are worthy alternatives to spectral methods. Electrochemical methods correspond to the nature of the antioxidant action associated with electron-proton transfer, and do not have the described limitations. The literature describes a few voltametric approaches to the study of antioxidant properties, based on the use of the DPPH^•^ as an oxidation model [9,10,11]. These approaches are based on determining the DPPH^•^ concentration or changing the magnitude of the reduction current after reaction with antioxidants. The approach also has a number of limitations: the method is quite sensitive to the material and surface of the indicator electrode and the measurement conditions; therefore, it can be implemented as an accessible and express method with low probability.

For the first time, this article will present new possibilities for using the DPPH^•^ as a signaling molecule of oxidant with potentiometric detection. The potentiometry method is devoid of the disadvantages, as described above. It is simple, accessible, express, and, in the future, can be easily implemented in a miniature version [12]. Therefore, the development of the potentiometric approach to the study of antioxidant properties using DPPH^•^ is quite promising.

It is known that the mechanisms of the biological action of antioxidants are varied, but from the point of view of chemical transformation, they can be reduced to three types of reactions: hydrogen atom transfer (HAT), electron transfer (SET), and chelation of metal ions of variable valence [13,14,15,16,17]. The concept, developed by us, is the creation of potentiometric methods, alternative to optical ones, for the study of antioxidant properties from the standpoint of various mechanisms of antioxidant (AO) action, both on the known models, traditionally used in the methods of studying AO properties, and on the models proposed by us for the first time, for the implementation of these tasks. For the successful implementation of this concept, the requirements for the models of oxidizing agent, used in the methods for studying AO properties, were formulated. Approaches to the potentiometric study of antioxidants, based on the reactions of electron and hydrogen atom transfer, have been successfully developed by our group for a long time [18,19,20,21,22,23]. Previously, we have shown that reactions with radical systems can be studied by the potentiometric method [20,21,22,23]. At the same time, this approach has a number of advantages associated with the absence of expensive equipment and activators of the analytical signal, for example, luminophore. For the first time, we proposed the hypothesis of a regular change in the potential during the reactions of peroxyl radical’s generation and inhibition ones. This regularity formed the basis for the approach using the radical generating system based on AAPH (2,2′-azobis(2-amidinopropane) dihydrochloride) [20,21,22,23]. The proposed new approach is based on the dependence of the potential change of radical systems in the process of reaction with an antioxidant by the hydrogen atom transfer mechanism, in an aqueous medium, coupled with the electron transfer process. Similarly, it can be assumed that the inhibition reaction of the DPPH^•^ radicals can also be studied by the potentiometry, since the main process in the reaction of DPPH^•^ with AO is electron-proton transfer. The purpose of this work is to develop the potentiometric method for studying radical reactions and the processes of their inhibition by antioxidants as an example of the reaction with the DPPH^•^ radical. Since reactions AO with DPPH^•^ can proceed by a combined mechanism with the formation of the stable Ox/Red pair DPPH^•^/DPPH-H [9,10,11], this gives grounds to hypothesize that this system is promising for use as a model in the Ox/Red potentiometry. This hypothesis opens up new possibilities for the potentiometry method both for studying the reactions of the DPPH^•^ radical with antioxidants and, in general, for studying radical reactions, which has not been previously described in the literature.

## 2. Materials and Methods

### 2.1. Apparatus

Voltammetric measurements were performed using the μAUTOLAB Type III potentiostat/galvanostat (Metrohm Autolab B.V., Utrecht, the Netherlands). A glassy carbon electrode (Metrohm Autolab B.V., Utrecht, the Netherlands) was used as a working electrode, a silver chloride (Metrohm Autolab B.V., Utrecht, the Netherlands) electrode was used as a reference electrode, and a glassy carbon rod (Metrohm Autolab B.V., Utrecht, the Netherlands) was used as an auxiliary electrode. 

Potentiometric measurements were carried out by using the pH-meter “Expert-pH” (LLC “Econics-Expert”, Moscow, Russia) with the function of measuring EMF and RS-232 interface. The measurements were taken by means of an EPV-1 redox-platinum electrode and EVL-1M silver-silver chloride electrode (Ag/AgCl/3 mol/dm^3^ KCl) (Gomelsky ZIP, Gomel, Belarus). 

Spectrophotometric measurements were carried out using an Evolution 201 UV-Visible Spectrophotometer (Thermo Fisher scientific, Waltham, MA, USA). The optical density of the solution DPPH**^•^** was measured at a wavelength of 517 nm in glass cuvettes l = 10 mm.

### 2.2. Reagents and Samples

The following reagents were used: 2,2-diphenyl-1-picrylhydrazyl, α-tocopherol, quercetin, (±)-catechin hydrate, and α-lipoic acid (Sigma-Aldrich, St. Louis, MO, USA). All other chemicals were of analytical reagent grade purity. Solutions of the antioxidant were prepared with ethanol. 

The tested plant material samples were commercially available (Pharmstandard, Moscow, Russia). Plant material sample solutions were prepared on daily basis, by accurate weighing of plant material samples and appropriate dilution in water and appropriate dilution in ethanol-water 40:60 (*v*/*v*).

Method for preparing water extracts: 3 g of dry plant material is extracted with 100 mL of boiling distilled water in a heat-resistant glass beaker for 15 min. Before measurements, the infusion was cooled to room temperature. Water-alcohol extracts were obtained by extracting 3 g of dry plant material with 100 mL of a water-alcohol mixture for 15 min.

### 2.3. Cyclic Voltammetry

A total of 9.9 mL of supporting electrolyte (tetrabutylammonium tetrafluoroborate (Bu4NBF4)) in solvent (ethanol, acetone, acetonitrile) was added in an electrochemical cell and CVs were recorded at potential range from 1.0 to 0.0 V and scan rate of 100 mV·s^−1^ (C(DPPH^•^) = 1 mmol/dm^3^, C(electrolyte) = 0.1 mol/dm^3^). Solutions of DPPH^•^ and the model antioxidant (quercetin) were prepared in solvents corresponding to those used as background ones. The antioxidant concentration varied in the range of (1–3)∙10^−4^ mol/dm^3^.

### 2.4. Spectrophotometric DPPH^•^ Assay

The spectrophotometric DPPH^•^ assay is based on the ability of the oxidized form of DPPH^•^ to absorb light at a wavelength of 517 nm [5,6]. The oxidized form of the radical has a deep violet color, while its reduced form decolorizes to a clear, pale-yellow solution. The amount of DPPH reacted is estimated from the reduction in radical uptake using a calibration curve (A = 0.1556·C(DPPH^•^)). The range of linearity of the calibration curve is (1–12)·10^−5^ mol/dm^3^. The analysis was carried out in glass cuvettes l = 1 cm; acetonitrile served as a reference solution.

## 3. Results and Discussion

**Scheme 1:**
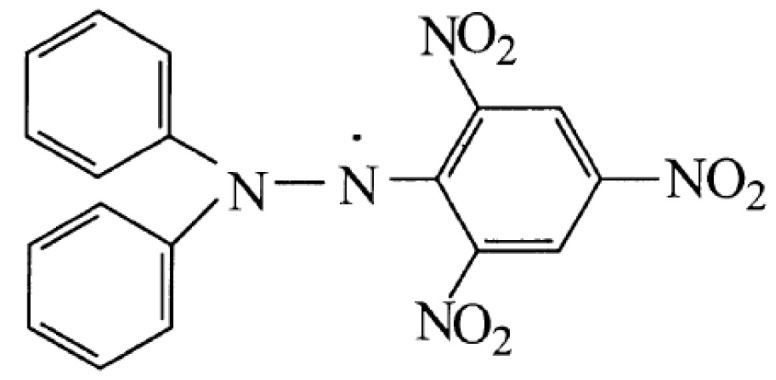
Structural formula of 2,2-diphenyl-1-picrylhydrazyl.

**Figure 1:**
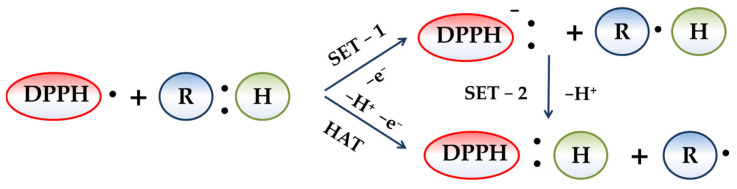
Transformations mechanism of the DPPH radical.

The structure of DPPH is as Scheme 1:

As noted previously, antioxidants react with DPPH^•^ stable radicals via a combined hydrogen atom transfer (HAT) and single electron transfer mechanism (SET). This fact also indicates the advantage of using the DPPH^•^ molecule in the study of the antioxidant/antiradical properties of substances [2,3,4,5,6]. The use of the molecule as a model in the reaction with AO makes it possible to measure the integral capacity, which simultaneously includes two mechanisms of antioxidant action in the body. The HAT reaction is a coordinated movement of a proton and an electron in one kinetic step (Figure 1). 

The free radical in the HAT-mechanisms intercepts one hydrogen atom from an antioxidant, and the antioxidant becomes a radical. The SET-reaction is realized by the one electron transfer from the nucleophile to the substrate with the formation of a radical intermediate, which can be involved in the further process, as shown in Figure 1. The antioxidant in SET-mechanisms gives up an electron to a free radical and becomes a radical cation. It is very difficult to distinguish between the HAT and SET reactions in such systems. In most cases, these two reactions proceed simultaneously, and the prevalence of a specific mechanism can be determined by antioxidant’s structure and solubility, the partition coefficient, and solvent polarity [24,25]. Reactions of DPPH^•^ with antioxidants in solution can proceed via mixed SET/HAT mechanisms (Figure 1).

Reactions between DPPH^•^ and secondary radicals (R^•^) and chemical reactions among R^•^ themselves (1) and (2) with the formation of recombination products are also possible in solution:

DPPH^•^ + R^•^ ↔ products (1)

R^•^ + R^•^ ↔ recombination products (2)

The reactions do not reflect the interaction of antioxidants with DPPH^•^ according to the described mechanisms of antioxidant action; however, these reactions make a significant contribution both to the study of stoichiometric ratios at various ratios of DPPH^•^ and antioxidant, and to the kinetic patterns of interaction. 

Therefore, the interaction of the DPPH^•^ with antioxidants proceeds according to the mechanisms underlying their transformation in the body, and is characterized by a high rate. Thus, DPPH^•^ satisfies a number of requirements for the model of an oxidizing agent used to study antioxidant properties [21]. Due to the fact that both mechanisms of the chemical transformation of the DPPH^•^ are associated with electron transfer, the use of the DPPH^•^ is quite promising in electrochemical studies, which was confirmed by the voltammetry [9,10,11].

### 3.1. Redox Behavior of the 2,2-Diphenyl-1-Picrylhydrazyl Radical

**Figure 2:**
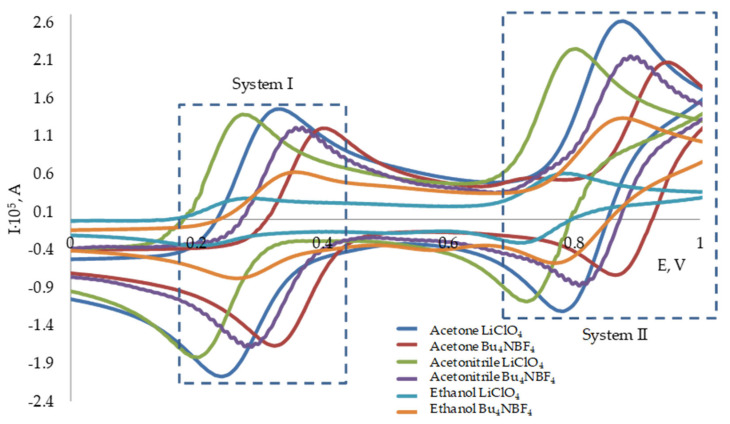
Cyclic voltammograms of 1 mM DPPH^•^ recorded on the glassy carbon electrode (solvent: ethanol/acetone/acetonitrile; electrolyte: 0.1 mol/dm^3^ Bu_4_NBF_4_/LiClO_4_; scan rate is 0.1 V/s).

The cyclic voltammetry was used to study the redox properties of the DPPH^•^ and to select a solvent and an electrolyte. Cyclic voltammograms of DPPH^•^ oxidation on a graphite electrode in various solvents are shown in Figure 2.

Table 1 presents the potentials and currents of DPPH^•^ reduction peaks obtained in various solvents and using various electrolytes.

Less pronounced reduction/oxidation peaks of the DPPH^•^ are observed in the ethanol; the peak currents height in the acetone and the acetonitrile is comparable. The lithium perchlorate (LiClO_4_) and the tetrabutylammonium tetrafluoroborate (Bu_4_NBF_4_) were used as the electrolyte. In this case, the introduction of tetrabutylammonium tetrafluoroborate as an electrolyte shifts the reduction peaks positions to a more positive region. 

Thus, acetonitrile and acetone are the most suitable solvents for electrochemical studies. At the same time, acetone is quite toxic; therefore, acetonitrile was used in potentiometric studies. Both lithium perchlorate and tetrabutylammonium tetrafluoroborate can be used as an indifferent electrolyte. 

Two systems can be observed on voltammograms. One system is located in the potential region of 0.20–0.40 V (system I); the second system is 0.72–0.94 V (system II). According to the literature data [26], system I corresponds to the electrochemical transformation according to Equation (3), system II corresponds to the transformation described in Equation (4).

(3) $${\left(\mathrm{Ph}\right)}_{2}N-N^{\cdot }-\mathrm{Ph}{\left({\mathrm{NO}}_{2}\right)}_{3}+e^{-}+H^{+}⇌{\left(\mathrm{Ph}\right)}_{2}N-\mathrm{NH}-\mathrm{Ph}{\left({\mathrm{NO}}_{2}\right)}_{3}$$

(4) $${\left(\mathrm{Ph}\right)}_{2}N^{+}=N-\mathrm{Ph}{\left({\mathrm{NO}}_{2}\right)}_{3}+e^{-}⇌{\left(\mathrm{Ph}\right)}_{2}N-\mathrm{NH}-\mathrm{Ph}{\left({\mathrm{NO}}_{2}\right)}_{3}$$

In most studies, the magnitude of the DPPH^•^ reduction peak in the system I is used as an analytical signal in voltametric studies [9,11]. In addition, there are studies in which the magnitude of the reduction peak in system II was used as an analytical signal [27]. 

It can be seen from the cyclic voltammograms and the data in Table 1 that DPPH^•^ reduction is quasi-reversible under these conditions. The anodic and cathodic peak-to-peak separations were found to be 80 mV and 60 mV, and the peak current ratio is close to 1 in both systems using the acetonitrile and the acetone. According to the data submitted by other authors [9,26], the DPPH^•^/DPPH-H system, when the experiment is carried out under other conditions, is reversible. This fact is significant enough, because it allows the use of the DPPH^•^/DPPH-H system in potentiometric studies, since the key in our approach is the presence of a reversible/quasi-reversible potential-determining system as an oxidizer model for obtaining an analytical signal in the potentiometry associated with the reaction between AO and the oxidizing agent. 

**Figure 3:**
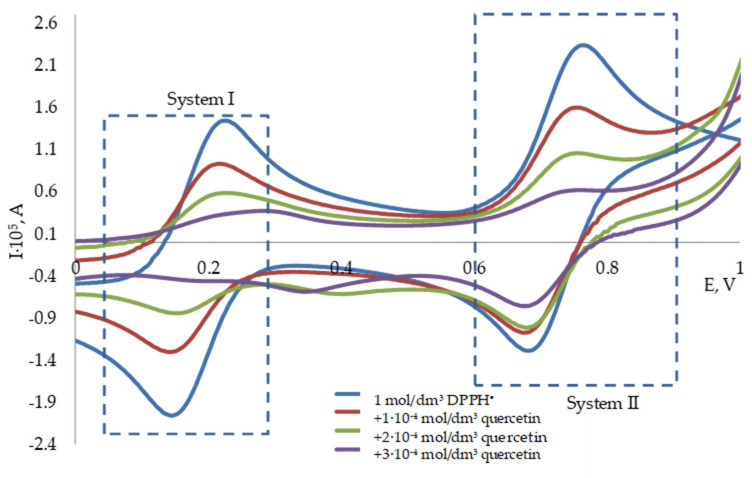
Cyclic voltammograms of 1 mol/dm^3^ DPPH^•^ with the quercetin at various concentrations ((1–3)∙10^−4^ mol/dm^3^) recorded on the glassy carbon electrode (solvent: acetonitrile; electrolyte is 0.1 mol/dm^3^ Bu_4_NBF_4_; scan rate is 0.1 V/s).

CVs on a glassy carbon electrode in the DPPH^•^ solution with the quercetin at various concentrations (C(DPPH^•^) = 0.1 mmol/dm^3^, acetonitrile, Bu_4_NBF_4_) are shown in Figure 3. The introduction of quercetin causes a regular decrease in the DPPH^•^ recovery peaks; I = −3.8·10^4^·C + 10.9 (R^2^ = 0.92) for system I, I = −2.7·10^4^·C + 14.7 (R^2^ = 0.95) for system II.

### 3.2. Potentiometric Investigation of the 2,2-Diphenyl-1-Picrylhydrazyl Radical

Previously, we have described and successfully applied a potentiometric approach have been study antioxidant properties using the Ox-Red components of the system on an example of the K_3_[Fe(CN)_6_]/K_4_[Fe(CN)_6_], for a wide range of objects [19,21,22,23]. However, the use of this approach for other models of oxidizing agents is difficult due to the inaccessibility of the Red component of the system, and consequently, the complexity of using two components of the system simultaneously. As shown earlier, the quasi-reversible process of oxidation/reduction DPPH^•^ makes it promising in potentiometric studies. For such cases, the approach with the second addition of an oxidizing agent to the electrochemical system was developed [28], and it was used with DPPH^•^. In the system, due to the excess of the oxidizing agent, after a chemical reaction with AO, the equilibrium between the oxidized DPPH^•^ and the resulting reduced form of DPPH^•^-H is established, which will act as a potential-determining system. 

Antioxidant additions to the DPPH^•^ solution will lead to a change in the potential as a result of the reactions in Figure 1. In this case, if there is an excess of the DPPH^•^ radical form in the solution, the dynamic equilibrium in the DPPH^•^/DPPH-H system will be established after a chemical reaction with antioxidants, and the system potential is described by Equation (5):

(5) $$E_{1}=E^{0}+b\cdot lg\frac{C_{DPPH•}-X}{X},$$

where $E_{1}$ is the potential of the DPPH^•^ solution, measured after the antioxidant addition, V; *C* (DPPH^•^) is the initial concentration of the 2,2-diphenyl-1-picrylhydrazyl, mol/dm^3^; and *X* is the antioxidant capacity—AOC_DPPH•_, mol-eq/dm^3^;

The system potential after the DPPH^•^ addition is expressed by Equation (6):

(6) $$E_{2}=E^{0}+b\cdot lg\frac{C_{DPPH•}-X+C_{DPPH•}^{\prime }}{X},\;$$

where E_2_ is the potential measured after the addition of the 2,2-diphenyl-1-picrylhydrazyl, V; $C_{\mathrm{DPPH}•}^{\prime }$ is the 2,2-diphenyl-1-picrylhydrazyl concentration in the first addition, mol/dm^3^; and *b* is the Nernst slope, equal to:

(7) $$b=2.303\cdot \frac{RT}{nF}$$

where *R* is the universal gas constant, 8.314 J/(mol·K); *T* is temperature, K; *F* is Faraday’s constant, 96,485 C/mol; and n is the number of electrons involved in the reaction.

Since the reduction of the DPPH^•^/DPPH-H system, as a system with a radical particle, can occur by a mixed mechanism, as well as in an organic solvent, the value constancy of the Nernst slope will most likely not be observed. The value of the Nernst slope is likely to be determined by the specific mechanism of the reaction with the antioxidant, which will depend on both the antioxidant nature and its concentration. Therefore, it is necessary to have information about the value of the Nernst slope under experimental conditions in order to correctly determine the antioxidant capacity. We have proposed the approach that allows in one experiment to obtain the value of the Nernst slope and the antioxidant capacity. It consists of an antioxidant supplement and two consecutive DPPH^•^ supplements. In this case, the calculation of the Nernst slope can be performed by introducing a second addition of the oxidizing agent and constructing a calibration curve against the background of the reaction with an antioxidant. The potential will be expressed by the equation after the introduction of the second DPPH^•^ additive:

(8) $$E_{3}=E^{0}+b\cdot lg\frac{C_{\mathrm{DPPH}•}-X+C_{\mathrm{DPPH}•}^{\prime }+C_{\mathrm{DPPH}•}^{\prime \prime }}{X},$$

where *E_3_* is the potential measured after the introduction of the third additive of the oxidizing agent, V, and $C_{\mathrm{DPPH}•}^{\prime \prime }$ is the DPPH^•^ concentration in the second additive, mol/dm^3^. 

The antioxidant capacity can be calculated by Equation (9):

(9) $$AOC_{DPPH•}=\frac{C_{DPPH•}\cdot \left(1-k\right)-k\cdot C_{DPPH•}^{\prime }}{\left(1-k\right)},\;$$

where $k=10^{\left(E_{1}-E_{2}\right)\;/b}$.

The equations system solution of Equations (5)–(8) allows you to find the Nernst slope and the antioxidant capacity. 

**Figure 4:**
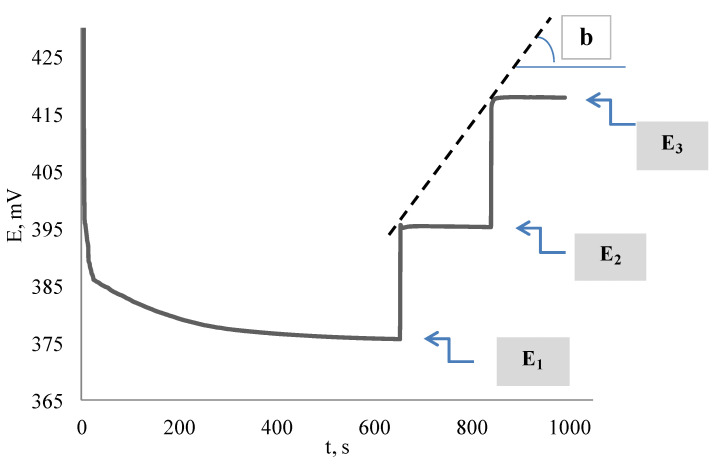
The dependence of the potential on time in introducing the 0.1 mmol/dm^3^ quercetin to the 1 mmol/dm^3^ DPPH^•^—E_1_; the 1 mmol/dm^3^ DPPH^•^ (${\;C}_{\mathrm{DPPH}•}^{\prime }$)—E_2_; and 3 mmol/dm^3^ DPPH^•^ (${\;C}_{\mathrm{DPPH}•}^{\prime \prime }$ )—E_3_.

The typical dependence of the potential on time in introducing the quercetin to the DPPH^•^ solution and the subsequent two additions of the DPPH^•^ is shown in Figure 4. 

**Figure 5:**
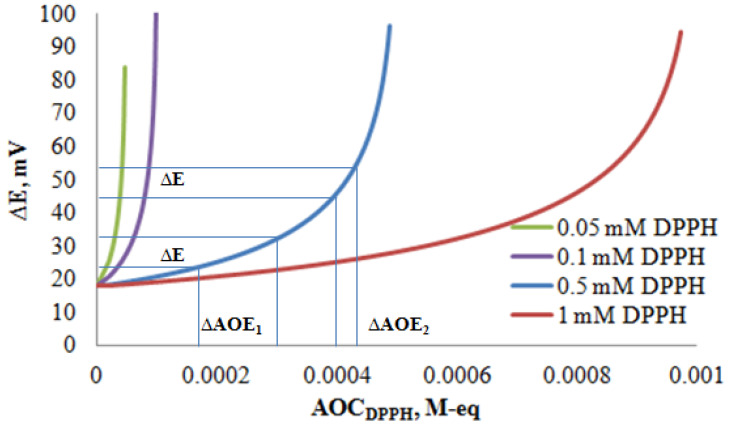
The dependence of the potential shift on the obtained AOC (ΔE is the determination error of the potential, ΔAOC_1_ is the determination error of the AOC in the region of gentle ascent, and ΔAOC_2_ is the determination error of the AOC in the region of steeper ascent).

Theoretical dependences of the registered deviation of the potential on the obtained values of the antioxidant capacity at various ratios of the DPPH^•^ were constructed to calculate the concentration intervals of the ratios of DPPH^•^ and antioxidant, which provide the smallest error determination. As illustrated in Figure 5, the smallest error determination of AOC_DPPH•_ will be observed in the region of steeper ascent of the dependence. In this case, the same potential difference leads to less pronounced deviations in the AOC in the region of the steeper ascent of the dependence. 

Thus, the operating ranges of AOC corresponding to smaller error for 0.05 mmol/dm^3^ DPPH^•^ are (1 to 4.8)·10^−5^ mol-eq/dm^3^; for 0.1 mmol//dm^3^ DPPH^•^, it is (4 to 9.8)·10^−5^ mol-eq/dm^3^; for 0.5 mmol/dm^3^ DPPH^•^, it is (2 to 4.9)·10^−4^ mol-eq/dm^3^; and for 1 mmol/dm^3^ DPPH^•^, it is (4 to 9.8)·10^−4^ mol-eq/dm^3^. 

**Figure 6:**
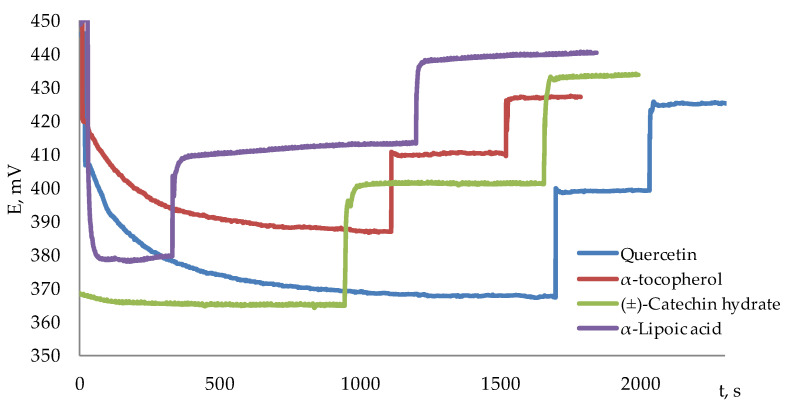
The dependences of the potential on time in introducing quercetin (0.7 mmol/ dm^3^), α-tocopherol (2.0 mmol/ dm^3^), (±)-Catechin hydrate (1.0 mmol/ dm^3^), and α-Lipoic acid (1.0 mmol/ dm^3^) to the DPPH^•^ solution $\left(C_{\mathrm{DPPH}•}^{}=1\;\mathrm{mmol}/{\mathrm{dm}}^{3},{\;C}_{\mathrm{DPPH}•}^{\prime }=1\;\mathrm{mmol}/{\mathrm{dm}}^{3},{\;C}_{\mathrm{DPPH}•}^{\prime \prime }=3\;\mathrm{mmol}/{\mathrm{dm}}^{3}\right)$.

Figure 6 shows the dependences of the potential on time in introducing quercetin, α-tocopherol, (±)-Catechin hydrate, and α-Lipoic acid to the DPPH^•^ solution, and two subsequent additions of the DPPH^•^. 

As can be seen from Figure 6, the kinetic dependences of the potential for all the studied antioxidants are subject to the expected classical patterns. This suggests that the developed approach can be used to determine the integral antioxidant capacity.

### 3.3. The Determination of the Antioxidant Capacity of Individual Antioxidants by Potentiometry and Spectrophotometry

Correlation studies of the capacity value, obtained by the proposed potentiometric and spectrophotometric approaches, using DPPH^•^ radicals, were carried out. The spectrophotometric method has been modified for the correct comparison of values. In the classic version of the spectrophotometric method, the results are expressed as equivalents of a standard compound, such as gallic acid, ascorbic acid, and quercetin. In the modified method, ${AOC}_{\mathrm{DPPH}•}^{\mathrm{spectr}}$ was determined by the decrease in the DPPH^•^ radical, i.e., the effective concentration of DPPH^•^ reacted with the antioxidant, according to the Equation (10), and was expressed in absolute units (mol-eq/dm^3^):

(10) $${\mathrm{AOC}}_{DPPH•}^{spectr}=(C_{DPPH•}^{0}-n\cdot C_{DPPH•}^{fin})\cdot m$$

where $C_{DPPH•}^{0}$ is the initial concentration of the DPPH^•^ radicals, mol/dm^3^; $C_{DPPH•}^{fin}$ is the final concentration of the DPPH^•^ radicals remaining after the reaction with the antioxidant, mol/dm^3^; n is the stoichiometric coefficient of reaction of AO with the DPPH^•^; m is the dilution of the DPPH^•^ solution after the antioxidant addition; and m is the dilution of the antioxidant solution. The stoichiometric coefficient was determined similarly by Equation (11):

(11) $$n=\frac{AOC_{DPPH•}}{C_{AO}}\;$$

Table 2 shows the antioxidant capacity and stoichiometric coefficients of the antioxidant in the reaction with the DPPH^•^ obtained by potentiometry and spectrophotometry. 

According to the data in Table 2, the results of the potentiometric determination in comparison with the known spectrophotometric approach are in good correlation with each other. The calculated *t*-test and F-test range from 0 to 4.5 and 2 to 14, respectively, which are significantly below the critical values at a 95% confidence level. This shows that the variances of the two populations are homogeneous. The average results are equal, and there is no significant difference in the accuracy of the potentiometric and spectrophotometric methods for determining the antioxidant capacity. 

However, the spectrophotometric method is significantly limited by the action of the Beer–Lambert law. In this regard, careful selection of the dilution of the object is necessary for the analysis of various samples. This issue is relevant, since the antioxidant properties of the compounds with respect to the DPPH^•^ radical depend significantly on the antioxidant concentration and on the ratio of AO/DPPH^•^ concentrations. In addition, the studied samples are often colored. In this sense, the potentiometric method has been an advantage; namely, it expands the range of determined concentrations, which spectrophotometry does not have. The determined value of AOE depends on the initial concentration of DPPH; in this study, the interval (1–94)·10^−5^ mol-eq/dm^3^ is presented. Table 3 presents the results of the antioxidant capacity in an extended range of concentrations. 

As expected, the concentrations ratio of the antioxidant and DPPH^•^ significantly affects the value of the stoichiometric coefficients of the reaction. Such analogies were also obtained by other researchers [29]. This is due to the fact that a change in the ratios of the antioxidant and DPPH^•^ can lead to a change in the reaction order, the limiting stage of the interaction process, and the intensification of side reactions. The literature describes similar data. They show a regular decrease of the stoichiometric coefficient of the antioxidant at a constant concentration of DPPH^•^ radicals, with an increase of the antioxidant concentration. This may be due to an increase in the contribution of side reactions (1) and (2) to the main reactions with the transfer of an electron/hydrogen atom from the antioxidant to the DPPH^•^. The ratio of antioxidant and DPPH^•^ concentrations is also a determining factor in the kinetic features of their interaction, leading to a decrease in the electron/hydrogen atom transfer rate constant with an increase of the antioxidant concentration. 

### 3.4. The Determination of the Antioxidant Capacity of Infusions of Medicinal Plants and Their Water-Ethanol Extracts

The conducted studies allow us to propose the developed approach for the analysis of complex objects, such as water and water-ethanol plant extracts. These objects were chosen as one of the most common objects containing a large number of exogenous antioxidants. The antioxidant capacity of infusions of medicinal plants and their water-ethanol extracts (*Calenduale flores*, *Herba Leonuri*, *Folia Betulae*, *Hyperici herba*, *Herbia Origanum vulgare*, *Menthae piperitae folia*, *Clitoria ternatea*, and *Hibiscus sabdariffa* L.) was determined by potentiometric and spectrophotometric approaches (Table 4). The relative standard deviation of the AOC values of complex objects does not exceed 9% by the potentiometric method.

It should be noted that the studied extracts are quite different in color. In this regard, it can be assumed that the color contribution can have a significant effect on the results obtained by the spectrophotometric method. However, this issue is practically not discussed in a large number of publications devoted to the spectrophotometric measurement of DPPH^•^ inhibition. 

**Figure 7:**
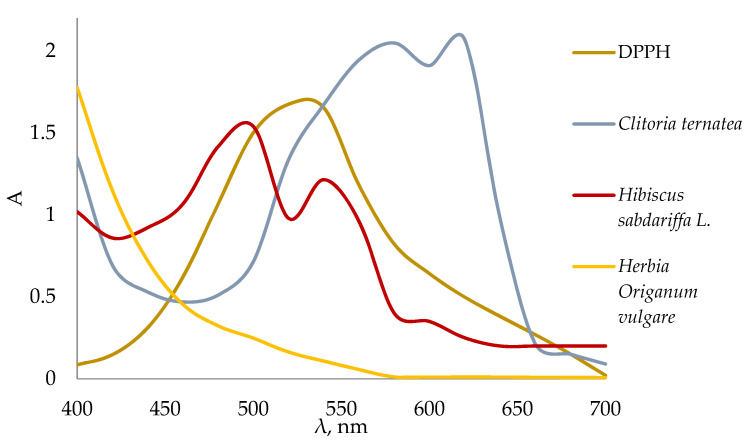
The absorption spectra of the 0.1 mmol/dm^3^ DPPH^•^ solution and water extracts of *Herbia Origanum vulgare*, *Clitoria ternatea*, and *Hibiscus sabdariffa* L.

Figure 7 shows absorption spectra of 0.1 mmol/dm^3^ DPPH^•^ solution and water extracts of *Herbia Origanum vulgare*, *Clitoria ternatea*, and *Hibiscus sabdariffa* L. 

**Figure 8:**
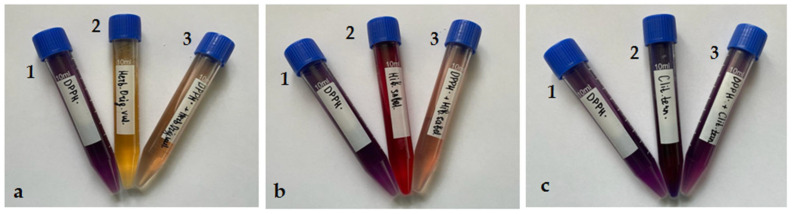
The photo of the 0.1 mmol/dm^3^ DPPH^•^ solution (1), test extracts (2), and mixture of DPPH^•^ and extract (3) ((**a**)—*Herbia Origanum vulgare,* (**b**)—*Hibiscus sabdariffa* L., (**c**)—*Clitoria ternatea*)).

A number of plant extracts are yellow to brown in color, as shown in Figure 8 on the example of *Herbia Origanum vulgare* (a(2)). 

There is no influence of the intrinsic color of the objects on the value of the optical density DPPH^•^ at 517 nm, as can be seen from the absorption spectrum. The results correlation, obtained by the potentiometry and the photometry, is 0.98 (R_crit_ = 0.67) for the extracts of *Calenduale flores*, *Herba Leonuri*, *Folia Betulae*, *Hyperici herba*, *Herbia Origanum vulgare*, and *Menthae piperitae folia*. 

However, red extracts (as an example *Hibiscus sabdariffa* L. in Figure 8b2) or blue extracts (as an example *Clitoria ternatea* in Figure 8c2) show absorption in the absorption maximum region of the DPPH^•^ at 517 nm (Figure 7). In this regard, the initial color of the extracts can make a significant contribution to the value of optical density measured at the wavelength. Table 4 shows that the AOC_DPPH•_ of the *Hibiscus sabdariffa L.* extract, determined by the spectrophotometric method, is significantly lower than the results of potentiometric measurements. 

The antioxidant capacity of *Clitoria ternatea* extracts could not be determined by the spectrophotometric method, because the test sample has an intense blue color. The decrease in optical density at 517 nm after the addition of *Clitoria ternatea* to DPPH^•^ was practically not observed. The AOC_DPPH•,_ determined by the potentiometric method, for water and water-ethanol extracts was (2.8 ± 0.2)·10^−2^ **mol-eq**/g and (3.6 ± 0.2)·10^−2^ **mol-eq**/g, respectively, which is consistent with the literature data on the high content of antioxidants in this sample [30]. 

Thus, the potentiometric method not only expands the boundaries of the determined concentrations, but has an undoubted advantage over the spectrophotometric approach in the analysis of objects with superposition of their own absorption spectra and on the absorption spectrum of the DPPH^•^. 

## 4. Conclusions

In the present work, it was shown that the DPPH^•^ stable radical molecule can be successfully used as a model of an oxidizing agent within our concept of creating potentiometric methods for studying antioxidant properties from the standpoint of various mechanisms of AO action. For the first time, new possibilities of using DPPH^•^ as a signal oxidant molecule with the potentiometric detection are shown. This opens up the possibilities of the potentiometry method for studying the interaction reactions of an oxidizing agent with an antioxidant by a mixed mechanism: electron transfer and hydrogen atom transfer. 

The CV method confirmed the presence of a quasi-reversible potential-determining system DPPH^•^/DPPH-H under experimental conditions. This fact makes it possible to use DPPH^•^ as the model of the oxidizing agent for obtaining an analytical signal by the potentiometry method. The proposed potentiometric approach consists in establishing an equilibrium between the oxidized DPPH^•^ and the reduced form of DPPH-H, formed after a chemical reaction with AO, which will act as a potential-determining system. The approach that allows obtaining the value of the Nernst slope and the antioxidant capacity in one experiment was proposed. It consists of an antioxidant supplement and two consecutive DPPH^•^ supplements. In this case, the calculation of the Nernst slope can be performed by introducing the second addition of the oxidizing agent and constructing a calibration curve against the background of the reaction with an antioxidant.

Solutions of individual antioxidants α-tocopherol, quercetin, (±)-catechin hydrate, and α-lipoic acid were studied by the developed approach. A high correlation with the results of spectrophotometric measurements is shown. At the same time, the potentiometry method is simple, accessible, and expressive; it significantly expands the concentration possibilities relative to the spectrophotometric method. It was shown that the spectrophotometry method has significant limitations in the extracts study of plant materials associated with the initial color of objects, which is typical for plant extracts, while the potentiometry method is devoid of these shortcomings. 

## Figures and Tables

**Table 1:** Potentials and currents of DPPH^•^ reduction peaks.

Solvent	Electrolyte	System I		System II		I_Red_/I_ReOx_	
		E_Red_, V	E_ReOx_, V	E_Red_, V	E_ReOx_, V	System I	System II
Ethanol	LiClO_4_	0.20	0.28	0.72	0.78	0.92	0.75
	Bu_4_NBF_4_	0.27	0.35	0.79	0.87	0.87	0.80
Acetone	LiClO_4_	0.24	0.32	0.79	0.87	1.11	0.95
	Bu_4_NBF_4_	0.32	0.40	0.87	0.94	1.12	1.05
Acetonitrile	LiClO_4_	0.20	0.27	0.72	0.79	1.13	0.98
	Bu_4_NBF_4_	0.28	0.36	0.81	0.88	1.11	0.93

**Table 2:** Antioxidant capacity with respect to DPPH^•^ radicals obtained by potentiometry and spectrophotometry (*n* = 5, *p* = 0.95).

AO	C(DPPH^•^), 10^−4^ mol/dm^3^	Introduced, 10^−5^ mol/dm^3^	Potentiometric Method			Spectroscopic Method		
			AOC_DPPH•_, 10^−5^ mol-eq/dm^3^	S_r,_%	n *	$AOC_{DPPH•}^{spectr},\;10^{-5}\;\mathrm{mol}-\mathrm{eq}/{\mathrm{dm}}^{3}$	S_r,_%	n *
α-tocopherol	1	2	4.30 ± 0.61	0.06	2.2	4.10 ± 0.41	0.04	2.1
	0.5	1	1.51 ± 0.22	0.05	1.5	1.71 ± 0.12	0.01	1.7
Quercetin	1	2.0	5.10 ± 1.19	0.09	5.1	5.08 ± 0.79	0.06	5.1
	0.5	1.0	2.40 ± 0.61	0.09	4.8	2.48 ± 0.16	0.08	5.0
(±)-Catechin hydrate	1	1.5	5.67 ± 0.48	0.03	3.8	5.47 ± 0.18	0.07	3.6
	0.5	1.5	3.02 ± 0.65	0.08	2.1	3.21 ± 0.09	0.07	2.1
α-Lipoic acid	1	1.0	2.42 ± 0.55	0.08	2.4	2.54 ± 0.06	0.04	2.5

n * is the stoichiometric coefficient of participation of the antioxidant in the reaction with DPPH^•^ (Figure 1, Equations (1) and (2)).

**Table 3:** Antioxidant capacity (AOC_DPPH•_) with respect to DPPH^•^ radicals obtained by the potentiometry (*n* = 5, *p* = 0.95).

AO	C(DPPH^•^), 10^−3^ mol/dm^3^	Introduced, 10^−4^ mol/dm^3^	AOC_DPPH•_, 10^−4^ mol-eq/dm^3^	S_r,_%	n *	b **	S_r_ (b)_,_%
α-Tocopherol	0.5	0.5	3.46 ± 0.25	0.03	6.9	0.033 ± 0.007	0.08
		1.0	2.63 ± 0.58	0.09	2.6	0.049 ± 0.009	0.11
		2.0	3.53 ± 0.14	0.02	1.8	0.053 ± 0.004	0.03
	1	3.0	6.63 ± 0.96	0.05	2.2	0.038 ± 0.007	0.07
Quercetin	0.5	0.7	3.90 ± 0.51	0.05	5.6	0.052 ± 0.006	0.06
		1.0	3.13 ± 0.14	0.06	3.1	0.051 ± 0.001	0.01
		1.5	4.90 ± 0.05	0.01	3.2	0.081 ± 0.009	0.07
	1	2.0	8.97 ± 0.58	0.02	4.5	0.028 ± 0.006	0.07
		3.0	9.40 ± 0.43	0.02	3.1	0.042 ± 0.005	0.05
(±)-Catechin hydrate	0.5	1.0	3.30 ± 0.25	0.03	3.3	0.073 ± 0.004	0.02
		2.0	4.10 ± 0.61	0.08	2.1	0.051 ± 0.008	0.10
	1	1.5	8.09 ± 1.25	0.07	5.4	0.037 ± 0.004	0.02
		2.0	4.10 ± 0.50	0.05	2.1	0.050 ± 0.006	0.08
α-Lipoic acid	1	1	3.72 ± 0.58	0.06	3.3	0.024 ± 0.002	0.02
	0.5	1	2.38 ± 0.51	0.08	2.4	0.030 ± 0.001	0.01
		0.7	1.72 ± 0.39	0.08	2.4	0.030 ± 0.006	0.05
		0.5	0.96 ± 0	0	2.0	0.040 ± 0	0

b ** is the Nernst slope in Equations (5)–(7); n *- stoichiometric coefficient (10).

**Table 4:** The antioxidant capacity of water and water-ethanol plant extracts obtained by the potentiometric (**AOC_DPPH•_**) and the spectrophotometric ($AOC_{DPPH•}^{spectr}$ ) methods (n = 5, *p* = 0.95).

Objects	Analyzed Extract	Potentiometric Method		Spectroscopic Method	
		AOC_DPPH•_, 10^−2^ mol-eq/g	S_r_	$AOC_{DPPH•}^{spectr},\;10^{-2}$ mol-eq/g	S_r_
Calendula (*Calenduale flores*)	Water	1.1 ± 0.1	0.06	1.1 ± 0.1	0.06
	Water-ethanol	1.2 ± 0.1	0.04	1.2 ± 0.2	0.08
Motherwort herb (*Herba Leonuri*)	Water	0.9 ± 0.2	0.10	0.9 ± 0.1	0.04
	Water-ethanol	2.4 ± 0.1	0.02	2.1 ± 0.3	0.06
Birch leaves (*Folia Betulae*)	Water	1.2 ± 0.3	0.09	1.2 ± 0.1	0.02
	Water-ethanol	2.8 ± 0.4	0.06	2.9 ± 0.2	0.03
St. John’s wort (*Hyperici herba*)	Water	1.9 ± 0.4	0.09	1.8 ± 0.4	0.09
	Water-ethanol	3.0 ± 0.4	0.05	2.9 ± 0.1	0.02
Oregano herb (*Herbia Origanum vulgare*)	Water	4.5 ± 0.4	0.04	4.2 ± 0.1	0.02
	Water-ethanol	10.2 ± 0.4	0.02	10.0 ± 0.1	0.02
Peppermint leaves (*Menthae piperitae folia*)	Water	2.7 ± 0.1	0.02	2.7 ± 0.1	0.02
	Water-ethanol	3.0 ± 0.1	0.02	3.1 ± 0.1	0.03
Hibiscus (*Hibiscus sabdariffa* L.*)*	Water	3.4 ± 0.2	0.05	2.6 ± 0.2	0.06
	Water-ethanol	4.2 ± 0.2	0.04	3.7 ± 0.2	0.03
Clitoria ternatea (*Clitoria ternatea*)	Water	2.8 ± 0.2	0.02	-	
	Water-ethanol	3.6 ± 0.2	0.02		

## Data Availability

All data generated or analyzed during this study are included in this article.
